# The Expanded Kinesin-13 Repertoire of Trypanosomes Contains Only One Mitotic Kinesin Indicating Multiple Extra-Nuclear Roles

**DOI:** 10.1371/journal.pone.0015020

**Published:** 2010-11-23

**Authors:** Bill Wickstead, Jamie T. Carrington, Eva Gluenz, Keith Gull

**Affiliations:** Sir William Dunn School of Pathology, University of Oxford, Oxford, United Kingdom; National Cancer Institute, United States of America

## Abstract

**Background:**

Kinesin-13 proteins have a critical role in animal cell mitosis, during which they regulate spindle microtubule dynamics through their depolymerisation activity. Much of what is known about Kinesin-13 function emanates from a relatively small sub-family of proteins containing MCAK and Kif2A/B. However, recent work on kinesins from the much more widely distributed, ancestral Kinesin-13 family, which includes human Kif24, have identified a second function in flagellum length regulation that may exist either alongside or instead of the mitotic role.

**Methodology/Principal Findings:**

The African trypanosome *Trypanosoma brucei* encodes 7 distinct Kinesin-13 proteins, allowing scope for extensive specialisation of roles. Here, we show that of all the trypanosomal Kinesin-13 proteins, only one is nuclear. This protein, TbKIN13-1, is present in the nucleoplasm throughout the cell cycle, but associates with the spindle during mitosis, which in trypanosomes is closed. TbKIN13-1 is necessary for the segregation of both large and mini-chromosomes in this organism and reduction in TbKIN13-1 levels mediated by RNA interference causes deflects in spindle disassembly with spindle-like structures persisting in non-mitotic cells. A second Kinesin-13 is localised to the flagellum tip, but the majority of the Kinesin-13 family members are in neither of these cellular locations.

**Conclusions/Significance:**

These data show that the expanded Kinesin-13 repertoire of trypanosomes is not associated with diversification of spindle-associated roles. TbKIN13-1 is required for correct spindle function, but the extra-nuclear localisation of the remaining paralogues suggests that the biological roles of the Kinesin-13 family is wider than previously thought.

## Introduction

The kinesin superfamily comprises a set of molecular motors that couple ATP hydrolysis to force production. This force is used by cells in the movement of various cargo along microtubules, and also in the regulation of microtubule dynamics. Within the superfamily as a whole, a number of distinct kinesin families can be determined on the basis of primary sequence of the protein motor domain. These families represent conserved groups of sequences which often have similar cellular functions (see [1]; [2]). The Kinesin-13 family contains microtubule depolymerases that have a critical role in animal cell mitosis (for review, see [3]). The family includes the protein MCAK (for mitotic centromere-associated kinesin) and also Kif2A and Kif2B, each of which localise to the spindle-poles and kinetochores or centromeres in mitotic cells [4]–[7]. Animal Kinesin-13 proteins at both these locations encourage microtubule disassembly [8] by ATP hydrolysis-dependent destabilisation of the microtubule end [9], and Kinesin-13 proteins play important mitotic roles in the regulation of spindle microtubule dynamics [10]–[12], the attachment of chromosomes to the mitotic spindle in metaphase [13]; [14], and the pole-ward movement of chromosomes in anaphase [15]; [16].

Most studies of Kinesin-13 function have concentrated on the subfamily of Kinesin-13 sequences containing Kif2A, Kif2B and MCAK, which has been referred to as the Kif2 or “animal-specific” subfamily [2]. This work has clearly established a mitotic role for several subfamily members. However, the Kinesin-13 family contains a much more widely distributed subfamily of proteins, Kinesin-13A [17], which includes human Kif24. This group probably represents the ancestral Kinesin-13 type, but much less is established as to the cellular function of proteins in this subfamily. Recent data on the action of the Kinesin-13A subfamily proteins, has identified a second flagellar function for Kinesin-13s, which may exist either alongside [18] or in place of [19]; [20] the function for Kinesin-13 proteins in the mitotic nucleus. This raises the possibility of other cellular roles for the more ubiquitous Kinesin-13 types, and suggests that extrapolating from the action of Kinesin-13B (MCAK/Kif2 subfamily) members to Kinesin-13A proteins such as Kif24 may not be appropriate.

The African trypanosome – causative agent of the neglected tropical disease of sleeping sickness – presents an especially interesting example of Kinesin-13 biology, since this protozoan parasite has a hugely expanded repertoire of distinct Kinesin-13A proteins encoded in its genome [21]. The cytoskeleton of the organism encompasses four separate sets of microtubules – cytoplasmic array (pellicular) microtubules, spindle microtubules, axonemal microtubules and those of the flagellar rootlet – which are nucleated from at least three spatially distinct microtubule organising centres. Moreover, although there has been an expansion of the Kinesin-13 family, there are no identifiable homologues for several other classes of kinesins involved in mitosis – including Kinesin-4, -5, -6, -7 and -8, family members [17]; [21]; [22]. These features raise the possibility that there has been either a diversification of mitotic functions in the trypanosome Kinesin-13 proteins, or a specialisation of roles for specific microtubule sets.

Here, we investigate the cellular roles of the Kinesin-13 proteins encoded by *Trypanosoma brucei*, looking in particular for activity in trypanosome mitosis. We demonstrate that, instead of additional mitotic functions, only 1 of the 7 encoded Kinesin-13 members is nuclear at any stage of the cell cycle. We demonstrate that this nuclear Kinesin-13 associates with the mitotic spindle, and that depletion of the protein results in defects in spindle regulation and chromosome segregation. These segregation defects are seen for both the large “housekeeping” chromosomes and a class of pathogenicity-associated small chromosomes which are believed to have a different mechanism of spindle-association. As well as this single mitotic Kinesin-13 sequence, we show that a second Kinesin-13 localises to the flagellar tip, but that the majority of the family members are cytoplasmic, playing an as yet uncharacterised role. These data shed light on the mechanisms of spindle regulation and mitosis in trypanosomes and also suggest that the cellular roles for Kinesin-13 members are likely to be broader than previously thought.

## Results

### Trypanosomes have a greatly expanded Kinesin-13 repertoire

**Figure 1:**
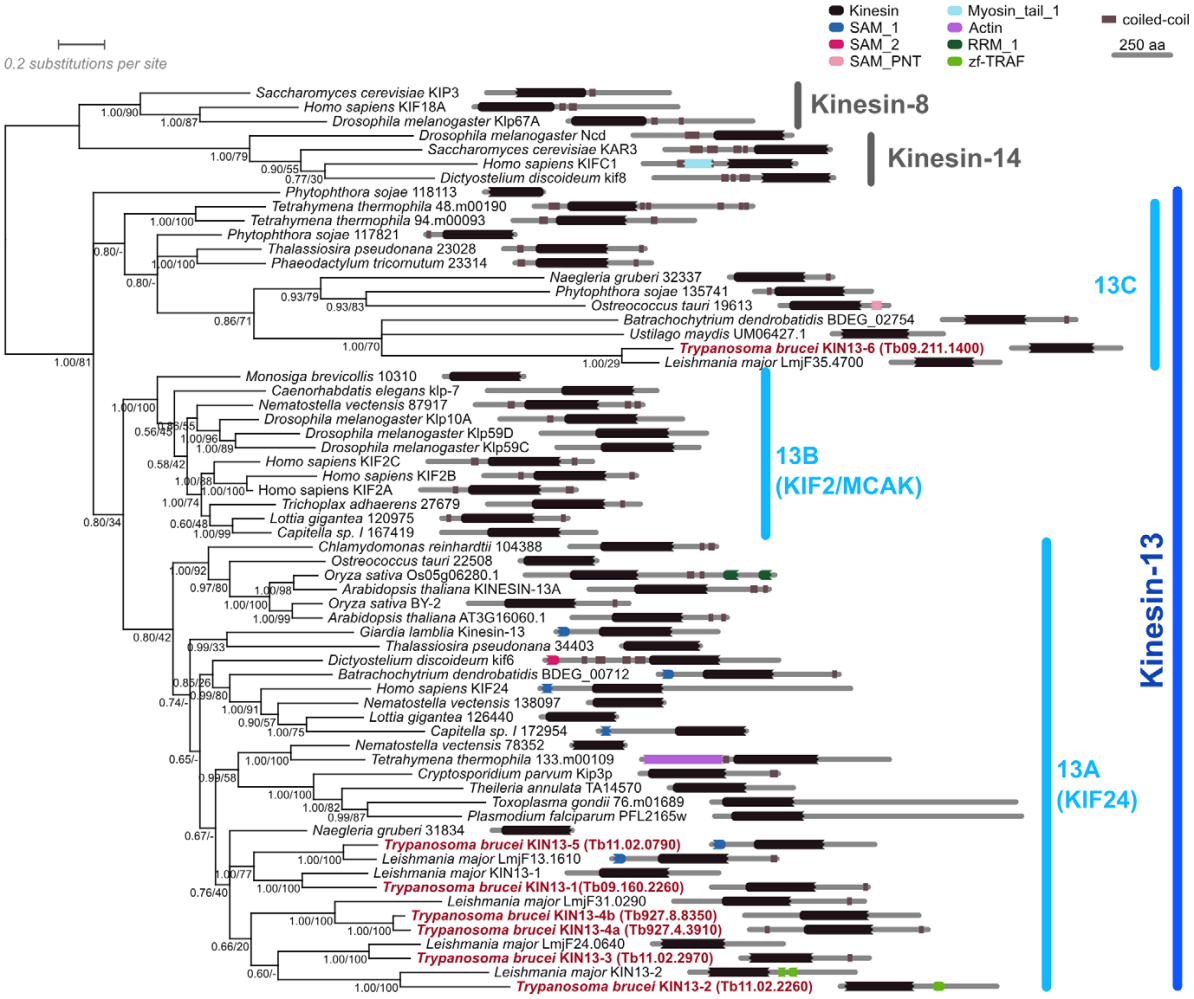
Phylogenetic analysis of Kinesin-13 family members from a diverse range of eukaryotes. The result of a Bayesian tree inference based on an alignment of the motor domain region is shown alongside protein architecture schematics showing the positions of predicted Pfam-A domains and coiled-coils (see Materials and Methods). The expanded *Trypanosoma brucei* repertoire is highlighted (red). A small number of Kinesin-8 and Kinesin-14 sequences are included as an outgroup. Numbers beside nodes represent support from Bayesian posterior probabilities or maximum likelihood bootstrap values (PP/BV).

The genome of *Trypanosoma brucei* encodes 7 proteins which can be bioinformatically classified as members of the Kinesin-13 family with good statistical support [17]. To more clearly define the relationships between these proteins and other kinesins, we extracted a set of representative Kinesin-13 sequences from a wide variety of eukaryotes from our previous analysis [17]. We analysed these by phylogenetic typing of their catalytic domains and on the basis of protein architecture (Fig. 1). These data show that six trypanosomal Kinesin-13 sequences belong to the largest and most widely distributed Kinesin-13 subfamily, Kinesin-13A. This subfamily may be the ancestral Kinesin-13 type [2]; [20], and contains the human kinesin Kif24, as well as the only Kinesin-13 proteins encoded by the single-celled organisms *Chlamydomonas* [19] and *Giardia* [18] (Fig. 1). We have named these proteins TbKIN13-1, -2, -3, -4a, -4b and -5 to follow on from the nomenclature of LmjKIN13-1 and LmjKIN13-2 used for 2 proteins studied in the related parasite *Leishmania major* [20]; [23] (note that similarly named proteins in *T. brucei* and *L. major* are orthologues).

All six trypanosome Kinesin-13A sequences appear to be the result of an ancient duplication of a single Kinesin-13 sequence in an ancestor of *Trypanosoma* and *Leishmania* (Fig. 1 and [17]). However, since duplication, the genes have diverged such that they now encode proteins with different architectures (Fig. 1) and are quite dissimilar at the primary sequence level (and average of 35% identity across aligned length). The repertoire of *T. brucei* has been further expanded by the relatively recent creation of two similar copies of KIN13-4 (KIN13-4a and KIN13-4b; 72% identity) accompanying the large-scale duplication of a ∼0.5 Mb region shared between chromosomes 4 and 8 [24]. Trypanosomes also encode a more divergent kinesin sequence (TbKIN13-6) predicted to be a member of the Kinesin-13C subfamily, which contains only sequences for which no functional data are available at this time. As expected, none of the trypanosomal sequences is a member of the Kif2/MCAK subfamily of kinesins (Kinesin-13B); no members of this subfamily have been found in organisms outside of the Holozoa (i.e. animals and their close relatives, such as choanoflagellates) [2]; [17].

All of the trypanosomal Kinesin-13 sequences contain the KVD-finger (necessary for microtubule depolymerization activity) and very highly conserved KEC motif (in the microtubule binding site) that are specific to this class of motors [25]; [26] (see Figure S1). This confirms our phylogenetic classification of these proteins as *bona fide* Kinesin-13 sequences, and also suggests (alongside a more general conservation of amino acids necessary for ATPase activity) that these proteins are biochemically similar to other Kinesin-13 sequences. In contrast, none of the Kinesin-13A or -13C sequences from any organism contain the positively-charged neck domain which precedes the catalytic domain in Kinesin-13B (“animal-specific”) subfamily proteins [27] and which regulates activity and localisation via Aurora B phosphorylation [28]–[30] (data not shown). This supports a previous suggestion that the neck domain is not a general feature of Kinesin-13 proteins outside of the Kinesin-13B subfamily [20].

### Trypanosomal Kinesin-13 proteins have at least 3 distinct cellular locations

**Figure 2:**
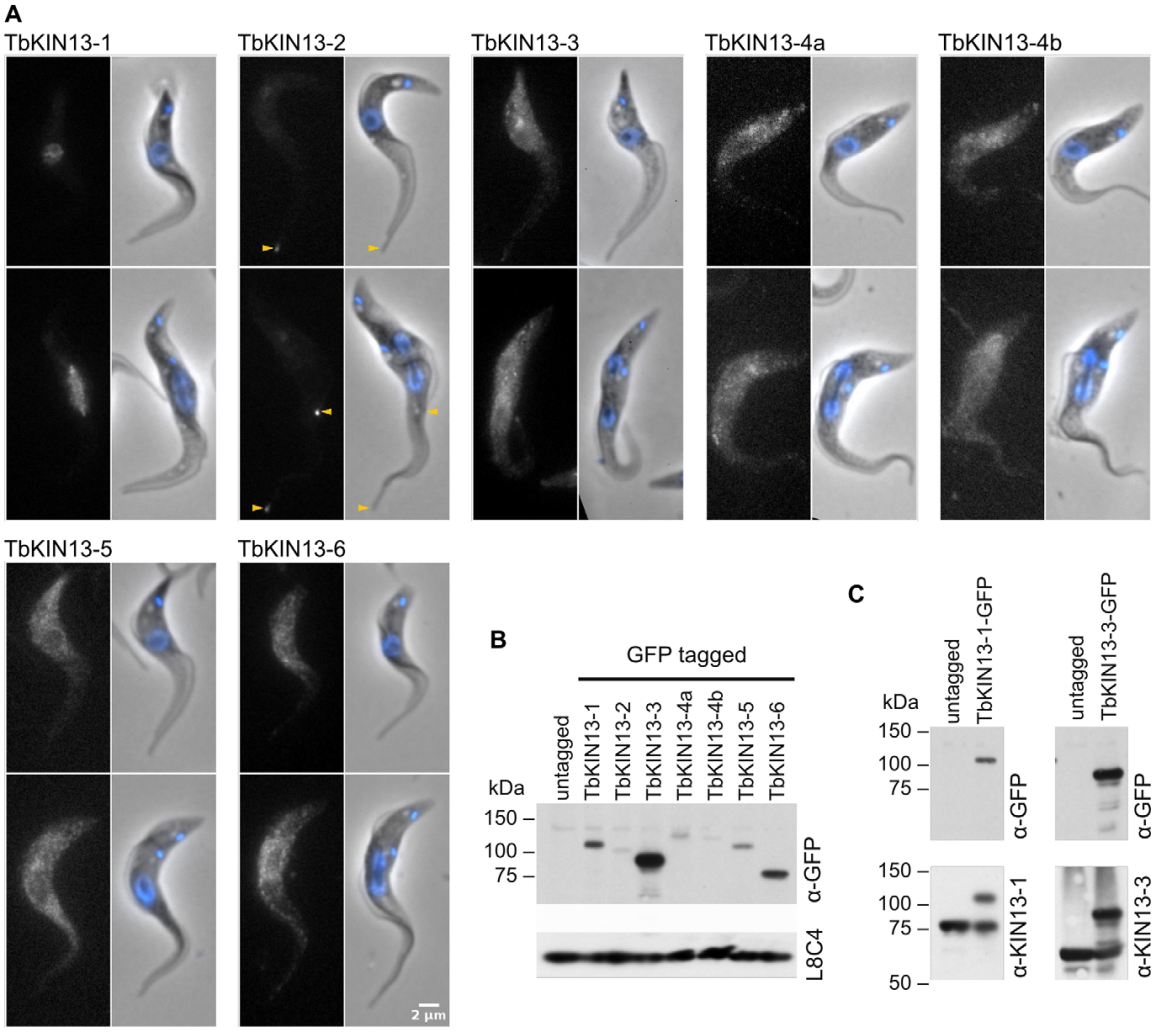
The cellular localisation of all seven *Trypanosoma brucei* Kinesin-13 family members. A) For each protein, direct fluorescence from C-terminal chimeras created by the insertion of *GFP* at the endogenous locus is shown (left panel), next to an overlay of signal from DAPI-stained DNA (blue) and phase-contrast image (right panel). Only TbKIN13-1 is nuclear. The accumulation of TbKIN13-2 at the flagellar tips is highlighted (arrows). All other Kinesin-13 family members in *Trypanosoma brucei* are found in the cytoplasm, or associated with the subpellicular array of microtubules. B) Levels of tagged protein in the cell lines seen in (A), demonstrated by Western blotting of whole cell lysates with α-GFP antibodies. Detection of the flagellar protein PFR2 by monoclonal antibody L8C4 [69], is shown as a loading control. C) Demonstration of near wild-type levels of protein expression produced by endogenous locus tagging with incorporation of endogenous UTRs (see Materials and Methods). Panels show whole cell lysates of cell lines containing TbKIN13-1-GFP or TbKIN13-3-GFP expressed from one of the two endogenous alleles, blotted with either α-GFP antibodies or affinity-purified polyclonal antisera against TbKIN13-1 or TbKIN13-3.

The most studied Kinesin-13 sequences are those from the 13B subfamily (which includes MCAK). These sequences are associated with the spindle during the open mitosis seen in animal cells (see for review [3]). Kif2A and Kif2B are associated with the spindle poles and also kinetochores in mitotic cells [5]–[7]; [31], whereas MCAK is seen at centromeres, spindle poles and the plus-ends of astral microtubules [4]; [32]. However, recent work on members of the ancestral 13A subfamily has revealed roles in the cilia/flagella of *Chlamydomonas*, *Giardia* and *Leishmania* [18]; [20]; [33]. To investigate the possible functions of the expanded trypanosomal Kinesin-13 family, we first localised each of the encoded proteins within the cell. We created C-terminal GFP-kinesin chimeras for each of the 7 sequences encoded in the *T. brucei* genome by insertion of *GFP* at the 3′-end of one endogenous locus of each coding sequence (see Materials and Methods for details). By incorporation of endogenous 3′ intergenic sequence into these tagging constructs, we created kinesin-GFP transgenes which were both transcribed by their endogenous promoters and also had endogenous UTRs in the mRNA produced, to ensure levels of expression similar to those of wild-type protein. The correct incorporation of the GFP tag at the 3′ end of the targeted kinesin coding sequence was checked by PCR (not shown) and immunoblotting of cell lysates with monoclonal antibodies directed against GFP (Fig. 2B). Each of these tagged proteins was localised by fluorescence microscopy in fixed cells taken from asynchronous cultures.

To our surprise, of the seven Kinesin-13 proteins encoded by trypanosomes, only one (TbKIN13-1) is found in the nucleus (Fig. 2A). This protein is orthologous to the protein LmjKIN13-1, which is found in the nucleus of *Leishmania major* [23], although with a different cell-cycle dependent distribution (see below). In contrast, TbKIN13-2 is highly enriched at the tip of the trypanosome flagellum (arrows, Fig. 2A). This localisation is consistent with the function of the orthologous leishmanial protein, LmjKIN13-2, which has been shown to regulate the length of the *Leishmania* flagellum in a manner which is dependent on domains involved in microtubule deploymerisation in other systems [20]. The remaining *T. brucei* Kinesin-13 proteins are all found predominantly in the cell body. Unlike TbKIN13-1, none of TbKIN13-2 to -6 are enriched in the nucleus at any stage of the cell-cycle, including during mitosis (Fig. 2A). Indeed, the localisations are consistent with active exclusion from this compartment.

**Figure 3:**
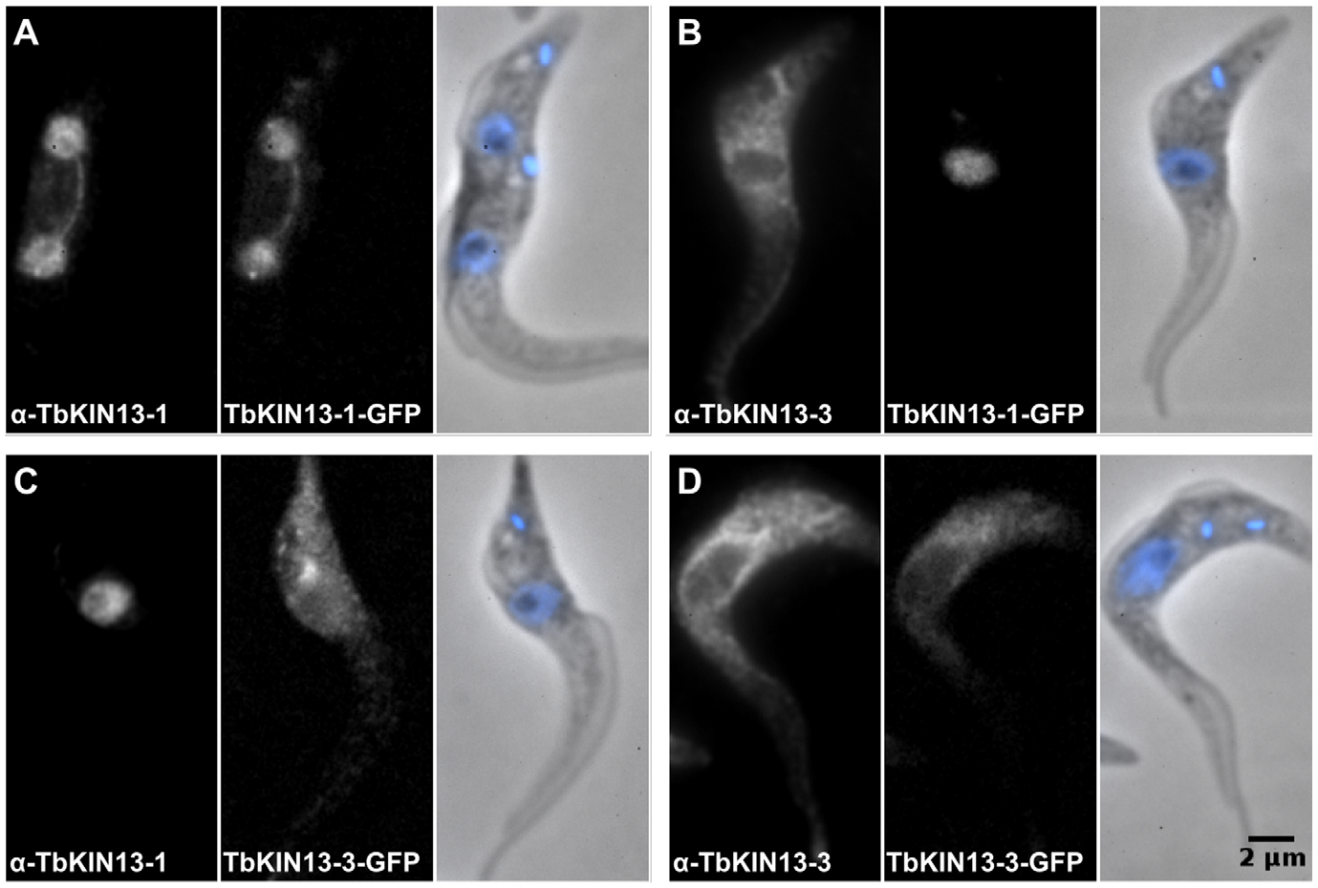
The subcellular locations of TbKIN13-1 and TbKIN13-3 are unaffected by C-terminal tagging with GFP. Images show signal from indirect immunofluorescence using affinity-purified polyclonal antisera against TbKIN13-1 or TbKIN13-3 (α-TbKIN13-1, α-Tb-KIN13-3) in cells expressing either TbKIN13-1-GFP or TbKIN13-3-GFP. Antibody and GFP co-localise when directed toward the same kinesin (A,D), but are specific to that kinesin (B,C). Both kinesins localise to the same cellular compartment whether tagged or untagged (A,C and B,D).

To confirm that the localisations of the trypanosomal Kinesin-13 proteins were not artefacts of the attachment of GFP, we selected 2 kinesins sequences – one nuclear (TbKIN13-1) and one cytoplasmic (TbKIN13-3) – which were expressed recombinantly and used to generate affinity-purified polyclonal antibodies. Immunoblotting of cell lysates from tagged cells shown that, as predicted, endogenous locus tagging with endogenous UTRs creates chimeric protein at levels very close to those seen for wild-type protein (Fig. 2C). The antibodies were specific to the kinesin against which they had been raised (Fig. 3 and Figure S2) and reacted strongly against either wild-type or GFP-tagged protein as expected (Fig. 3). Moreover, they showed the same localisation for TbKIN13-1 and TbKIN13-3 (nuclear and cytoplasmic, respectively) in cells containing either tagged or untagged protein (Fig. 3), demonstrating that the localisation of the GFP-tagged proteins was representative of that for the wild-type protein. These data show that levels of the C-terminally GFP-tagged kinesins are very close to untagged levels and that both tagged and untagged proteins have the same cellular localisation. Together with the ability to use the endogenous-locus tagging approach to localise other proteins to their known cellular locations (not shown), these data indicate that the finding of only TbKIN13-1 in the nucleus of *T. brucei* is representative of the true cellular distribution of these proteins.

### TbKIN13-1 associates with the spindle, but is not cell-cycle regulated

**Figure 4:**
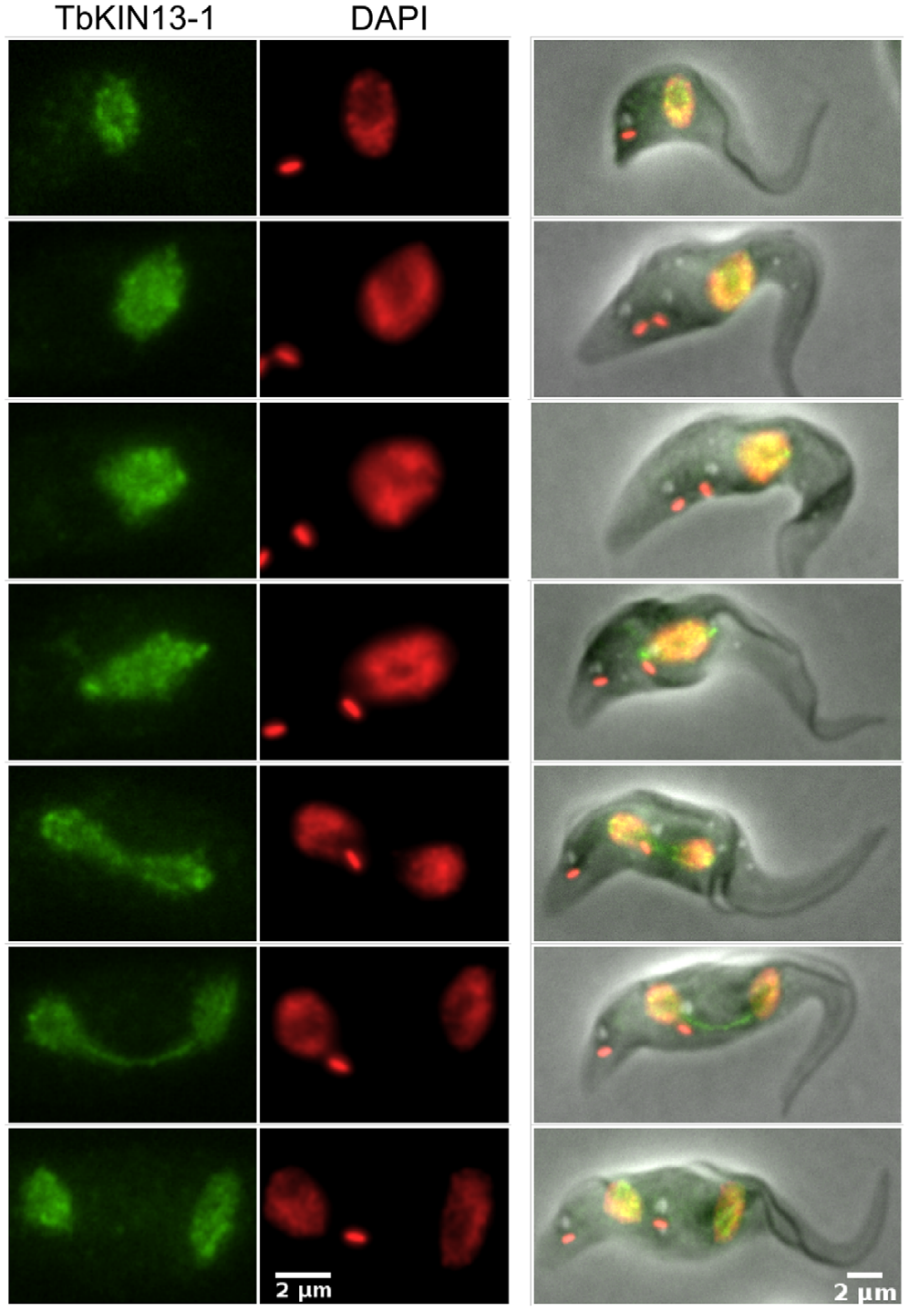
TbKIN13-1 is found in the nucleus throughout the cell-cycle. Direct fluorescence signal from TbKIN13-1-GFP (green) and DAPI-stained DNA (red) are shown for representative cells at different stages of the cell-cycle (ordered from G1 to cytokinesis, top to bottom). All images were captured and processed with the same settings, such that fluorescence levels can be directly compared.

Dubessay et al. [23] have previously shown that in *Leishmania major*, a Kinesin-13 family member which is the orthologue of TbKIN13-1 shows a cell-cycle-dependent regulation of protein levels that may be the result of proteasome activity. Strikingly, however, this cell-cycle-dependent regulation is not a feature of TbKIN13-1. The *T. brucei* protein is present in the nucleus throughout the cell cycle and at consistent levels (Fig. 4). The protein is distributed throughout the nucleoplasm, even in cells undergoing mitosis. This consistent localisation and lack of level variation is not an artefact of protein tagging, since the same result was obtained when using antibodies directed against untagged TbKIN13-1 (not shown).

**Figure 5:**
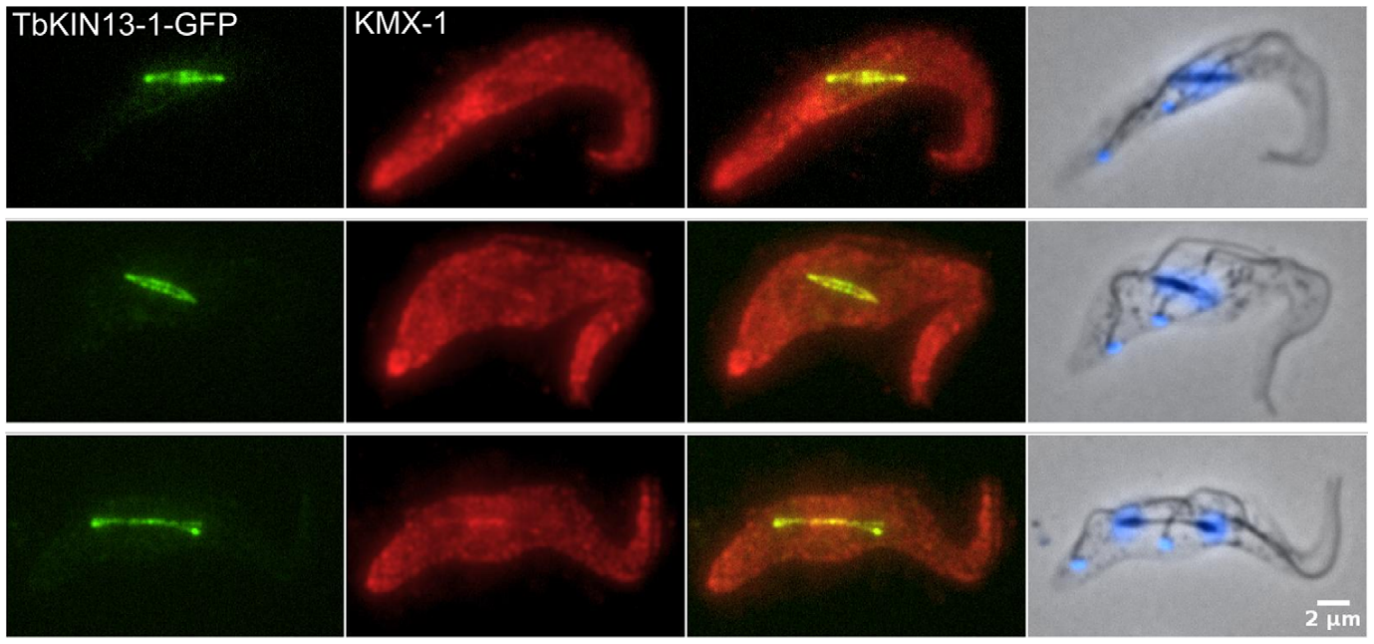
TbKIN13-1 is associated with the mitotic spindle. Direct fluorescence signal from TbKIN13-1-GFP (green), and indirect immunofluorscence using the anti-β-tubulin monoclonal antibody KMX-1 [67] (red) for ‘cytoskeletons’ made by extracting cells with non-ionic detergent in microtubule-stabilising buffer. Overlays of DAPI-stained DNA (blue) and phase-contrast images are also shown (right panel).

The consistent levels of TbKIN13-1 in the nucleoplasm do not, of course, preclude this protein having a specific role in the mitotic spindle. To test whether there are biochemically distinguishable pools of Kinesin-13 within the constant levels of TbKIN13-1 in the trypanosome nucleus, we assessed the subnuclear localisation of TbKIN13-1 in cells that had been detergent-extracted under conditions which favour microtubule stability (cytoskeleton preparations). Under these conditions, proteins freely diffusing in the nucleoplasm are removed to the soluble fraction, leaving only proteins that are tightly associated with either chromatin, the nucleolus or the spindle. When cells expressing TbKIN13-1-GFP are treated in this way, the nucleoplasmic signal is removed, to reveal a pool of TbKIN13-1 which is associated with the mitotic spindle (Fig. 5). All cells containing an identifiable spindle also contained detergent-insoluble TbKIN13-1 distributed across the spindle in an even manner. Conversely, in cells with no mitotic spindle, no signal remained in the nucleus following detergent treatment (not shown). Noteably, the TbKIN13-1 spindle-associated pool does not form a distribution expected for a component of kinetochores; there is no punctate staining identifiable on spindles – neither on diamond-shaped spindles characteristic of early mitosis (which are thought to represent metaphase), nor on later anaphase spindles (Fig. 5).

Consistent with the non-nuclear localisation observed for TbKIN13-2 to -6, none of these proteins was found associated with the the mitotic spindle in cytoskeleton preparations of cell lines carrying tagged versions of these proteins (not shown).

### Knockdown of TbKIN13-1 causes defects in spindle dynamics

**Figure 6:**
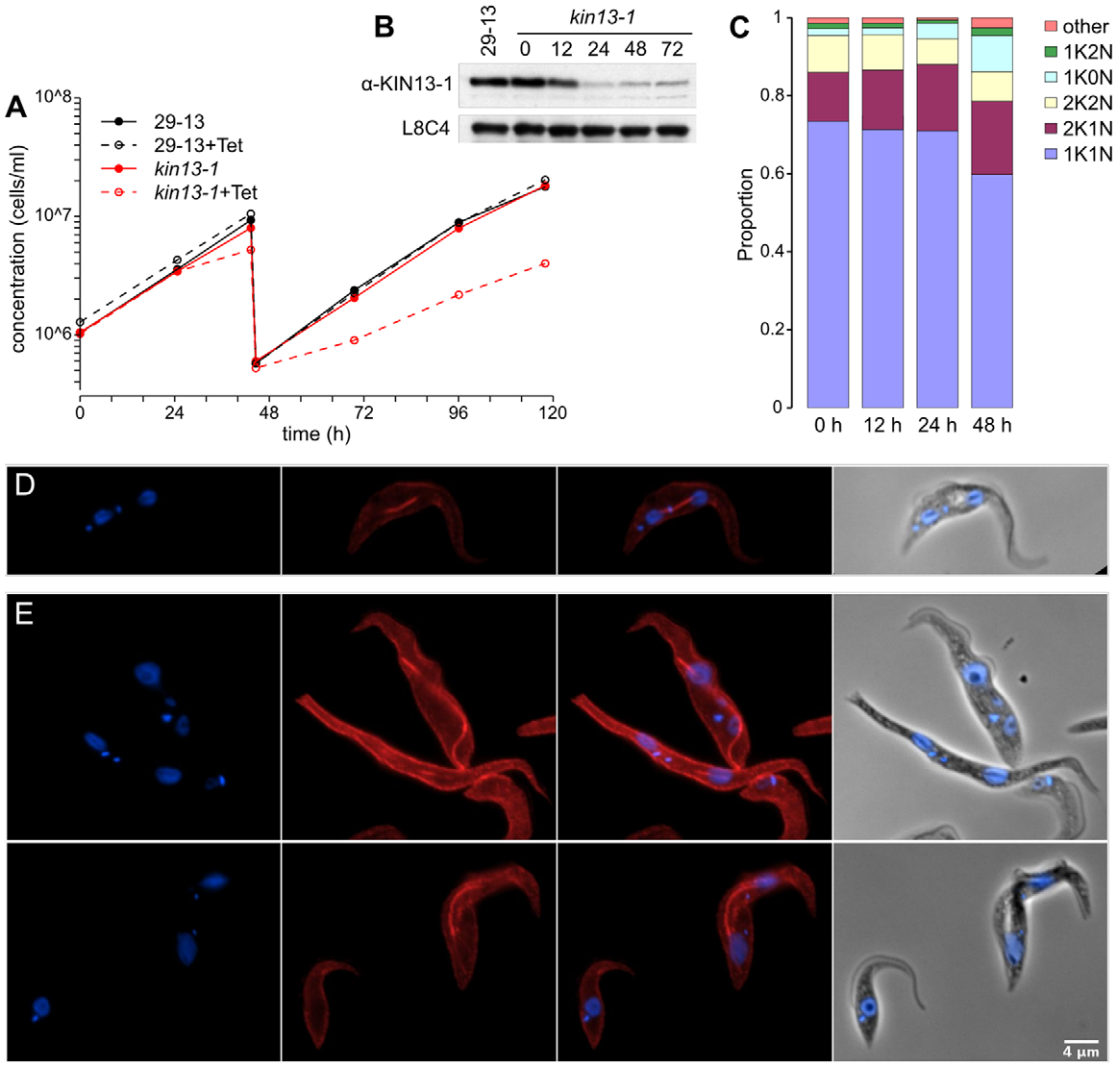
Induction of RNA-interference against *TbKIN13-1*. A) Cell growth is reduced 24 h after the addition of tetracycline. B) Levels of TbKIN13-1 are similar in parental cells line (29-13) and RNAi cells before induction (0) but rapidly fall at post-induction (12, 24, 48, 72 h shown), as seen by Western blot using affinity-purified anti-TbKIN13-1 polyclonal antibody (α-KIN13-1). Monoclonal antibody L8C4 which detects a flagellar protein [69] was used as a loading control. C) Induction of RNAi is accompanied by an increase in anuclear ‘1K0N’ cells (K = kinetoplast, N = nucleus; each time point represents 500 cells). Indirect immunofluorescence using anti-β-tubulin monoclonal antibody KMX-1 [67] (red) and DAPI (blue), before (D) and after (E) induction shows that RNAi results in cells with unevenly segregated nuclear DNA and abnormal tubulin distribution.

To investigate the role of TbKIN13-1 in trypanosome cells, we generated a stable transgenic cell line, in which RNA interference (RNAi)-mediated depletion of TbKIN13-1 could be induced by the addition of tetracycline. Induction of RNAi in these cells results in a persistent reduction in growth rate after 24 h (Fig. 6A), which is preceded by a strong reduction in the levels of TbKIN13-1 in cells (Fig. 6B). We further investigated this growth defect by assessing the position in the cell cycle of induced cells. The progression of *T. brucei* cells through the cell cycle can be monitored using as markers the duplication of the DNA of the single mitochondrion (which forms a structure known as the kinetoplast) and the segregation of the the nuclear DNA. At the start of the cell cycle, trypanosomes have one kinetoplast (K) and one nucleus (N). Under normal conditions, the kinetoplast divides first resulting in 2K1N cells, which later go through mitosis (2K2N), and finally, cytokinesis to create two daughter 1K1N cells. Surprisingly, depletion of TbKIN13-1, although causing a growth phenotype, does not greatly alter the distribution of cells in the different portions of the cell cycle, as assessed by the numbers of kinetoplasts and nuclei within each cell (Fig. 6C). However, there is a significant increase in the proportion of cells in a 2K1N configuration, from 12±1.6% before RNAi to 19±1.9% 48 h post-induction (p = 0.009 for overall change in 2K1N; χ^2^) which are in G2 or early mitosis [34]. There is also an increase in anucleate cells, termed ‘zoids’ [35] (1K0N; <2% of the population at 0 h; 9±1.4% at 48 h post-induction), which are the result of failures in mitosis or cytokinesis in trypanosomes (p<0.001 for overall change; χ^2^).

In spite of this relatively small effect on organelle numbers, there is a much more dramatic alteration in cell morphology at later points following RNAi induction. Many of these cells possess what appear to be grossly long microtubule structures within the cell (Fig. 6E), which are most likely spindles that have not disassembled correctly. These long microtubule based structures seen in cells at these later time points following depletion of TbKIN13-1 are almost certainly spindle-derived, since 3D deconvolution microscopy shows them to be separate from the subpellicular microtubules under the cell surface, and instead associated with nuclear DNA at least some point along their length (Figure S3). In agreement with this, thin-section transmission electron microscopy of cells after RNAi induction reveals bundles of intra-nuclear microtubules similar to those seen in anaphase (Figure S4), but no abnormal microtubule bundles are observed elsewhere in the cells.

**Figure 7:**
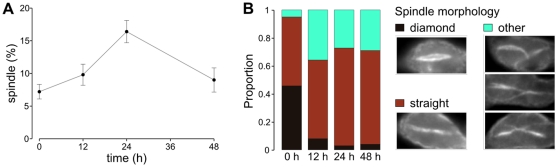
Effect of RNAi against *TbKIN13-1* on spindle formation. Knockdown of *TbKIN13-1* results in a rapid accumulation in cells undergoing mitosis (A; as judged by the presence of a mitotic spindle). However, there is a concomitant decrease in the proportions of these spindles with a ‘diamond’ configuration characteristic of cells in early mitosis and an increase in abnormal spindle morphologies (B). All time points represent counts of 500 cells or spindles.

The proportion of cells with a mitotic spindle increases dramatically upon induction of RNAi against *TbKIN13-1*, before any effect on growth rate is apparent (Fig. 7A). However, the morphology of these spindles alters greatly; there is a rapid decrease in the proportion of mitotic cells with ‘diamond’ shaped spindles that are characteristic of early mitosis and an increase in the ‘straight’ spindles seen during anaphase, as well as the appearance of aberrant spindle types (Fig. 7B). At later time-points (48 h post-induction and beyond), as cell morphology is more grossly affected, it becomes increasingly difficult to unambiguously identify cytoplasmic microtubule bundles as being true spindles, leading to an apparent reduction in the proportion of spindles in the population (Fig. 7A). Given that these microtubule bundles are most likely spindles that have failed to depolymerise, it is likely that this is a significant underestimate of the number of spindle-derived structures which are in the population at these times. Importantly, the rapid, early reduction in the proportion of diamond-shaped spindles in cells is of a higher magnitude than can be accounted for by the increase in spindle numbers generally at these early time points (Fig. 7) and must therefore represent a loss of these spindle types as well as an accumulation of other types.

### TbKIN13-1 is necessary for the segregation of all chromosomal classes

The genome of *T. brucei* is unusual in possessing a very large number (∼100) of small linear chromosomes which carry genes encoding major surface proteins used by the parasite for immune evasion (see [36]). These chromosomes constitute ∼10% of the total nuclear DNA. In spite of their great number, each small chromosome is segregated with great fidelity at mitosis [37]; [38] by an interaction with the mitotic spindle that is different from that of the larger chromosomes [39]; [40]. The molecular nature of this interaction is still unclear and it is possible that the interaction of the minichromosomes with the spindle is independent of a discrete centromere [41]. Given this different mechanism of segregation, spindle proteins which depolymerise pole-to-kinetochore microtubules in a similar manner to that of MCAK in animal cells, would be expected to have a differential effect on the mitotic movement of large chromosomes and minichromosomes.

**Figure 8:**
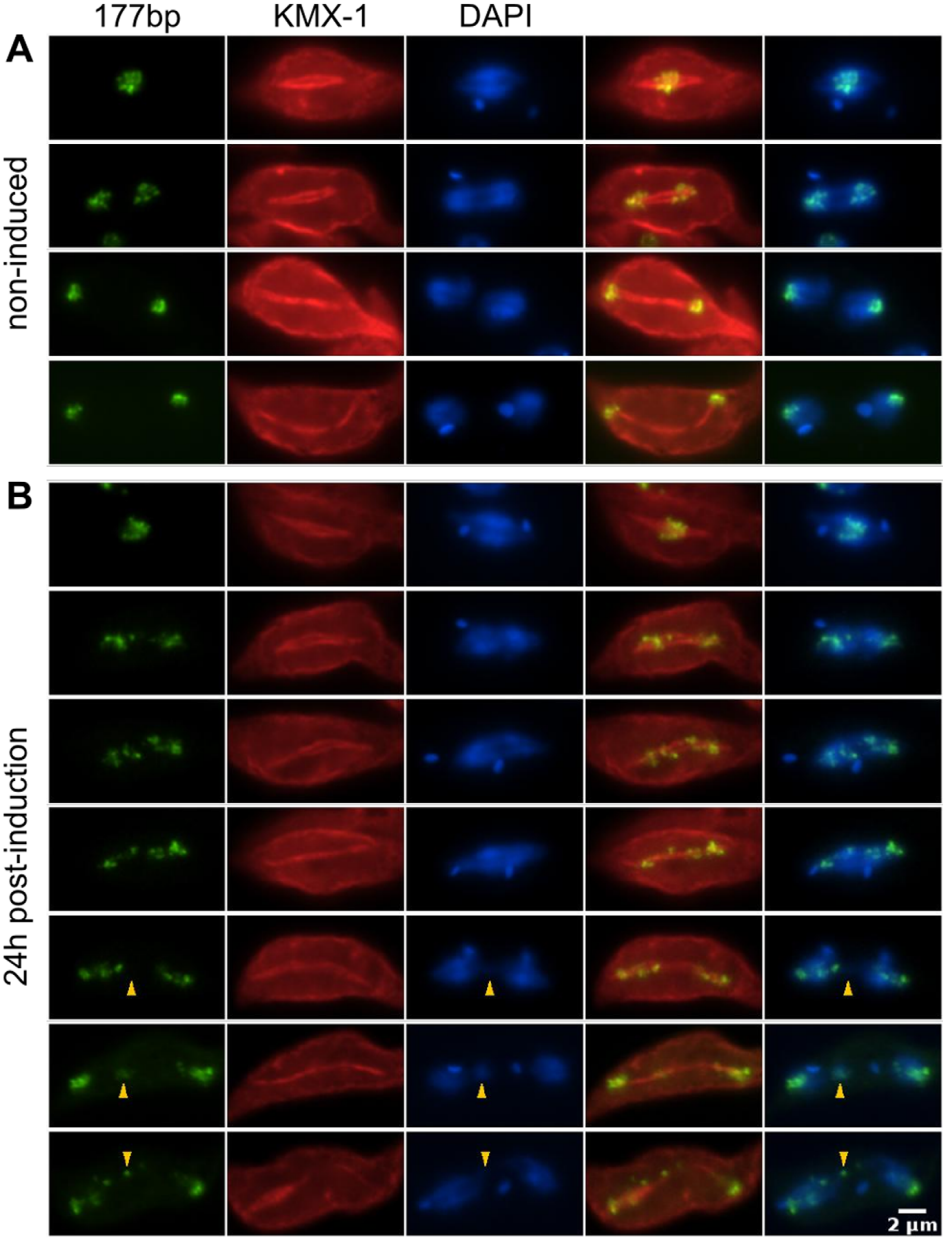
Knockdown of *TbKIN13-1* causes defects in the segregation of both large and mini-chromosomes. Shown is signal from *in situ* hybridisation using a 177 bp repeat specific to the mini-chromosomal population (green) and combined immunofluorescence using anti-β-tubulin monoclonal antibody KMX-1 [67] (red). DAPI (blue) shows total DNA. In non-induced cells, minichromosomes progress rapidly to the spindle poles at the onset of anaphase, where they remain (A). Following induction of RNAi against *TbKIN13-1*, there are defects in the movement of both the small chromosome population and bulk DNA (B). Arrows highlight lagging DNA in anaphase cells.

We assessed the effects of knockdown of TbKIN13-1 on the movement of trypanosome chromosomes during the early stages of RNAi-mediated depletion. A bulk DNA stain was used as a proxy for the large chromosomes, since they represent 90% of this signal. Minichromosomes were marked by fluorescence *in situ* hybridisation to a 177 bp repeat, which is specific to this chromosome class (and the 5 intermediate-sized chromosomes). In the early stages of mitosis, a diamond-shaped spindle forms in the trypanosome nucleus around which minichromosomes cluster in a manner that is highly reminiscent of the metaphase plate formed in animal cells ([39]; Fig. 8A). At the onset of anaphase, minichromosomes move rapidly to the spindle poles at which they remain until the spindle is disassembled. The larger chromosomes, as viewed by either bulk DNA (Fig. 8A) or individual chromosomal loci [39], move slightly behind these small chromosomes.

When levels of TbKIN13-1 are depleted by induction of RNAi for 24 h, congression of minichromosomes to the spindle equator occurs in an apparently normal manner (Fig. 8B). However, coherent movement to the poles at anaphase is disrupted, with cells showing instead minichromosomal signal at other positions along the spindle. In these cells, bulk DNA movement has also been affected. Signal representing lagging chromosomes can also be seen, both from regions of the nucleus that stain for minichromosomes and also from those which do not (arrows, Fig. 8B). These data show that the segregation at anaphase of both the multiple small chromosomes and also the larger centromeric chromosomes of *T. brucei* are dependent on TbKIN13-1 function.

## Discussion

In the above, we have investigated the expanded Kinesin-13 family found in the protozoan parasite *Trypanosoma brucei*. Of the 7 distinct Kinesin-13 family members encoded in the genome, we show that only one, TbKIN13-1 (Tb09.160.2260), is nuclear. This protein is found in the nucleoplasm throughout the cell cycle, but associates with the mitotic spindle when it forms. Depletion of the protein causes aberrations in spindle formation and function – with defects in the anaphase movement of both the large “housekeeping” chromosomes and the numerous specialised minichromosomes.

### Kinesin-13 proteins in trypanosome mitosis

The nature of mitosis in trypanosomes is of biological interest for a number of reasons. First, the trypanosome genome encodes few identifiable homologues of components of the mitotic apparatus characterised in yeast or vertebrate cells [21]. This may reflect the greater evolutionary divergence between trypanosomes and these models than between yeast and humans, but may also be a consequence of important differences between the systems. Second, the nuclear genome consists of 11 large diploid chromosomes and ∼100 small linear chromosomes (mostly minichromosomes, plus 1–7 “intermediate-sized” chromosomes; see [36]). Each of the minichromosomes is segregated with great fidelity at mitosis [38] via an interaction with the spindle [39]. However, the mitotic spindle contains only ∼30 microtubules [42], meaning that a canonical pole-to-kinetochore microtubule interaction cannot exist for each minichromosome. Moreover, although the large chromosomes contain repetitive centromeres, minichromosomes are insensitive to etoposide-mediated cleavage, leading to the suggestion that minichromosomes may be acentric [41]. RNAi-mediated depletion of the cohesin complex component SMC3 also resulted in a differential effect, in which the separation of large chromosomes was disrupted with no apparent problems in minichromosome segregation [43]. Thirdly, *T. brucei* lacks several important spindle motors/depolymerases, including any members of the Kinesin-4, -5, -6, -7 and -8 families [21]; [22]. This has lead to the hypothesis that diversification of function in the expanded Kinesin-13 family might provide some of the functions played by these kinesins in other systems [21].

The data presented here add significantly to the relatively sparse molecular information on trypanosome mitosis. Although TbKIN13-1 associates with the spindle, the protein does not have a localisation consistent with a discrete association with centromeres as is the case for MCAK [4]; [32]. In cytoskeleton preparations, TbKIN13-1 signal is seen on a spindle as a whole; there is no detectable enrichment of the protein near the spindle equator during chromosome congression at metaphase, or apparent concentration at the spindle poles. However, depletion of TbKIN13-1 by RNAi produces lagging chromosomes – raising the question as to whether TbKIN13-1 is functioning on all spindle microtubules or only a subset. For Kinesin-13B subfamily members, for example, depletion of Klp10A by RNAi disrupts turnover of all spindle microtubules [44]. In contrast, knockdown or inhibition of MCAK specifically affected turnover in pole-to-kinetochore microtubules without an apparent effect on spindle microtubules more generally [45]; [46]. Our finding that TbKIN13-1 functions in the segregation of both large chromosomes and the numerous minichromosomes, combined with the rapid alteration in spindle morphology on RNAi-mediated knockdown and the localisation of TbKIN13-1, are all indicative of a primary function for this protein in regulation of spindle dynamics generally, rather than a specific role in the small number of pole-to-kinetochore microtubules in the trypanosomal mitotic spindle.

Finally, the finding that only 1 of the 7 Kinesin-13 family members in *T. brucei* is nuclear is strong evidence against the hypothesis that different Kinesin-13s might have specialised functions in the mitotic spindle. This might imply that the mitotic spindle of trypanosomes is simpler in terms of its repertoire of molecular motors than many other systems. However, there is still a strong possibility that some of 18 kinesins encoded in the *T. brucei* genome that cannot be grouped into previously defined families [17] might also play a role. Interestingly, the localisation of TbKIN13-1 is similar to those of two trypanosomal kinesin-like proteins that interact with the chromosome passenger complex (CPC) [47] – the Aurora B/Ipl1-containing complex which plays a key role in detecting incorrectly oriented kinetochores in metaphase (reviewed in [48]). Neither of these kinesin-like sequences can be confidently grouped into any of the known families of kinesins [17]; [22]: one is an ungrouped kinesin, whilst the other has little similarity to *bone fide* kinesins and is very unlikely to be a true motor. Nonetheless, both proteins are present in the nucleoplasm before and after mitosis [47] and associate with the mitotic spindle, again with no apparent enrichment in kinetochore-like structures. However, in dramatic contrast to the effects of TbKIN13-1 depletion, knockdown of these kinesin-like proteins results in a huge decrease in the number of observed spindles. This contrasting effect of depletion is presumably more a reflection of the role of the CPC in spindle formation in trypanosomes, rather than evidence for any direct antagonism between TbKIN13-1 and CPC-associated kinesins.

### Differences between orthologues in *Leishmania* and *Trypanosoma*

TbKIN13-1 is orthologous with the protein LmjKIN13-1 of *Leishmania major*. The function of LmjKIN13-1 in mitosis has not been directly assessed, but its localisation in cells over-expressing tagged protein has been analysed [23]. The cellular distribution of LmjKIN13-1 differs from that of TbKIN13-1 in that its expression is markedly regulated during the cell cycle, probably by proteasomal degradation [23]. This regulation is dependent on a minimal region of 60 aa in the C-terminal tail which is also required for nuclear accumulation. A similar region is present in TbKIN13-1 (42% similar), but our data show that TbKIN13-1 protein levels are not cell-cycle regulated. TbKIN13-1 is found in the nucleoplasm throughout the cell cycle at reasonably constant levels, and this is true of tagged or native protein. Hence, the mitotic function of TbKIN13-1 is not dependent on cell-cycle-specific degradation.

LmjKIN13-2 has been functionally characterized and shown to act as a microtubule depolymerase with a role in the regulation of flagellar length [20]. The localisation of TbKIN13-2 at the flagellar tip is entirely consistent with a similar function in *T. brucei*, and its cellular function was not further investigated here. However, the localisation of TbKIN13-2 is much more discrete than that of tagged LmjKIN13-2, which was found all along the flagellum and also in the cell body in addition to a modest enrichment at the flagellar tip [20]. These differences may be the consequence of genuine differences between the distributions of the wild-type proteins in these different organisms, but it should also be considered that they are as likely the result of the different methods of expression: the LmjKIN13-2 protein was over-expressed from a multicopy episome with ectopic processing sequences, whereas TbKIN13-2 in this study used integration of tag at the endogenous locus and reconstituted the endogenous UTRs of the mRNA.

### Diversification of function in the Kinesin-13 family

The Kinesin-13 family is divided into 3 subfamilies – 13A, 13B and 13C. Kinesin-13B – which contains Kif2A, Kif2B and MCAK – is by far the most studied subfamily. However, the most widely distributed family is 13A, which contains sequences from animal, plant, basal fungi and also diverse protozoan species. It is likely that this is the ancestral family of Kinesin-13 sequences from which the “animal-specific” (more properly holozoan-specific) 13B family arose. This is also reflected in the levels of support for the subfamilies. In the analysis presented here, as in others [17]; [22], support for monophyly of Kinesin-13B is very strong (see Fig. 1), but support for a single Kinesin-13A clade is weaker (posterior probability of 0.8). This is consistent with the idea that the Kinesin-13A subfamily is ancestral to the Kinesin-13B group, since such a relationship would mean that the Kinesin-13A subfamily is not truly monophyletic (to the exclusion of Kinesin-13B). It is, however, clearly a useful categorisation for class of Kinesin-13 sequences which are bioinformatically distinct from the holozoan Kinesin-13B subfamily. In contrast, the subfamily 13C is less well supported than either 13A and 13B in this analysis and should be treated with some caution until such time as further support for its existence is found, since it may reflect only a loose grouping of more divergent Kinesin-13 proteins, rather than a true monophyletic class (see Fig. 1).

Recent findings of functions for Kinesin-13A members outside of the mitotic spindle [18]–[20], raise the significant question as to what cellular role we should expect in other Kinesin-13A proteins, such as human Kif24, which have yet to be functionally characterised. Interestingly, unlike for 13B (Kif2/MCAK), there is no clear consensus emerging from the data currently available. As discussed above, LmjKIN13-2 functions in flagellar length regulation [20], whereas we have shown here the TbKIN13-1 has a role in mitosis. Depletion of *Chlamydomonas Cr*Kinesin-13, which is the only Kinesin-13 encoded in this organism [22], inhibits flagellar assembly and disassembly with no reported defects in cell division [19]. On the other hand, in *Dictyostelium*, which possesses no flagellum/cilium, disruption of the gene encoding Kif6 (Kinesin-13A) results in mitotic defects including spindle malformation and lagging anaphase chromosomes [49]. The single Kinesin-13 protein encoded in *Giardia* seems to encompass both these roles, since ectopic expression of a rigor mutant causes both mitotic defects and also flagellar elongation [18].

It is likely that the ancestral roles of the Kinesin-13 family may have included at least two functions at disparate cellular locations: one on the mitotic spindle and a second at the tip of the flagellum, with the uniting feature being the depolymerising action of these proteins. This second, flagellar function may perhaps provide the evolutionary rationale behind the fact that the chytrid fungus *Batrachochytrium dendrobatidis* has retained a copy of Kinesin-13A when this family has been lost from most fungi – since this chytrid retains the ability to build flagellate zoospores [50]. Interestingly, gene duplication in the lineage that gave rise to trypanosomes has lead to the specialisation of these roles into 2 distinct proteins, which in organisms such as *Giardia* are found in a single gene [18].

However, our data suggest that even this division between mitotic and flagellar functions is not the full extent of Kinesin-13 function. While TbKIN13-1 and TbKIN13-2 play these roles, the remaining 5 sequences are found in locations inconsistent with such a role. In *Arabidopsis*, Kinesin-13A is associated with the distributed Golgi stacks found in the plant cells [51]. None of the trypanosomal Kinesin-13 proteins specifically localise to the single Golgi apparatus, which is found on the posterior side of the nucleus near the point at which the flagellum exits the cell [52]; [53], but these data point to roles for Kinesin-13 outside either the spindle or flagellum. It is also noteworthy, of course, that even within the 13B subfamily, Kif2A has important biological roles in (non-dividing) neurons [54]–[56]. One of the interesting features of the expansion of Kinesin-13A in trypanosomes is that all the members probably originated from a single gene in an ancestor of this lineage. Our phylogenetic analysis shows that the first duplication event separated TbKIN13-1-like sequences from a TbKIN13-2-like group, neatly dividing up possible mitotic and flagellar functions. However, more recent duplications within both these groups (TbKIN13-1/-5 and TbKIN13-2/-3/-4, respectively) have resulted in proteins which have neither localisation. These events are still quite old, predating the split of *Leishmania* and *Trypanosoma* ∼250 Mya [57], but they do demonstrate a surprising plasticity in Kinesin-13 function.

As yet, we do not know the cellular role played by all of the non-nuclear Kinesin-13 sequences in trypanosomes, and this will be the basis for further investigation. However, the work presented here demonstrates that only one of the expanded Kinesin-13 repertoire is nuclear and that this protein functions in the unusual mitosis of the trypanosome. It is also suggestive of a broader set of roles for Kinesin-13 family members than has previously been thought.

## Materials and Methods

### Bioinformatic analysis and phylogenetics

The sequences of all Kinesin-13 proteins encoded in the genomes of 45 diverse eukaryotes were extracted from the dataset available from a recent comprehensive analysis of kinesin superfamily phylogeny [17]. This set was reduced to the representative organisms seen in Fig. 1 and trimmed to the regions containing the motor domain based on the Pfam “Kinesin motor domain” model, PF00225 [58]. Sequences were aligned using MAFFT6.24 [59] adopting the E-INS-i strategy, and trimmed to well-aligned blocks (446 characters). A Bayesian phylogeny was inferred from the protein alignment using metropolis-coupled Markov chain Monte Carlo (MCMCMC) method as implemented in the program MrBayes3.1.2 [60]. Two independent runs of 800,000 generations were performed from random start trees, using the WAG substitution matrix with a gamma-distributed variation in substitution rate approximated to 4 discrete categories (shape parameter estimated from the data). Support for the inferred topology was generated from 100 bootstrap replicates of the data using PhyML3.0 [61].

### Cell lines and culture

Endogenous-locus tagging was performed using procyclic-form *Trypanosoma brucei* Lister 427 cells [62]. For stable inducible RNA interference, the transgenic 29-13 cell line [63] which expresses tetracycline repressor and T7 polymerase, was used. Cells were maintained as axenic cultures in SDM79 at 28°C, as described by Brun and Schönenberger [64].

### Endogenous-locus tagging and RNA interference

A construct for specific C-terminal tagging of trypanosome endogenous loci, pEnG0, was generated from the endogenous locus plasmid pEnT5-G [65], by replacing the *TY-GFP-TY* gene (including flanking UTR sequences) with *GFP* coding sequence flanked by multi-cloning sites upstream and downstream. Briefly, pEnT5-G was digested to completion with MluI and partially with SphI, and the 4.6 kb fragment encompassing the *HYG* gene and bacterial processing sequences was selected. Into this construct was placed a sticky-end linker generated by annealing the primers (CGCGAATTCACTAGTGTAGATCTGAACTCTAGAGGATCCTCGAGAGCTCATG and AGCTCTCGAGGATCCTCTAGAGTTCAGATCTACACTAGTGAATT). A *GFP* coding sequence was then reintroduced by amplification from pEnT5-G with the primers (gc agatct GTGAGCAAGGGCGAGG and gc tctaga tta CTTGTACAGCTCGTCCATG), and insertion between the BglII and XbaI sites of the above plasmid. Map and full sequence for the pEnG0 plasmid are available from www.billwickstead.net/vectors.shtml.

Targeting sequences followed the schema for integration at endogenous gene loci outlined in [65]. Briefly, PCR amplicons containing ∼200 bp from the 5′-end of the 3′-UTR and ∼200bp from the C-terminal end of the CDS of interest were cloned together into the SpeI-BglII sites upstream of the *GFP* in pEnG0, such that the C-terminal end of the CDS was in frame with *GFP*. In the same step, a NotI linearisation site was introduced between the 3′-UTR and CDS. Following this step, the full 3′ intergenic region between the gene of interest and the endogenous downstream gene (amplified by PCR) was introduced downstream of *GFP* using 2 sites from the small multi-cloning site present in this location. Integration of these constructs at the targeted endogenous locus results in transgenic lines in which one allele of the CDS of interest contains *GFP* at its C-terminus, but both 5′- and 3′-UTRs are identical to untagged copy. Vectors were linearised by digestion with NotI restriction endonuclease and transfected into procyclic-form Lister 427 cells as described previously [63], followed by selection of stable transformants with 25 µg ml^−1^ hygromycin (Sigma-Aldrich). Correct integration was assessed by diagnostic PCR from genomic DNA of clonal transformants (not shown) and also immunoblotting of cell lysates separated by SDS-PAGE against a mixture of two anti-GFP monoclonals (7.1 and 13.1; Roche). Primers used for vector construction are available in Table S1.

Stable inducible RNAi against *TbKIN13-1* was performed in 29-13 cells [63] by transfection with a plasmid derived from p2T7-177 [66] by the introduction of a gene fragment encompassing nucleotides 1387–1833 of TbKIN13-1 (Tb09.160.2260; amplified by PCR from genomic DNA) between the inducible T7 promoters. This vector was linearised with NotI restriction endonuclease and transfected into procyclic-form 29-13 cells as described previously [63]. Stable transformants were selected with 5 µg ml^−1^ phleomycin (Sigma-Aldrich). After selection of resistant lines, cells were removed from selection 72 h before the induction of RNAi by the addition of 1 µg ml^−1^ tetracycline (Sigma-Aldrich) to normal growth medium.

### Generation and purification of polyclonal antisera

Coding sequence fragments encoding residues 466–691 of TbKIN13-1 (Tb09.160.2260) and 322–566 of TbKIN13-3 (Tb11.02.2970) were amplified by PCR from *T. brucei* TREU927 genomic DNA and cloned in frame into the bacterial expression vector pQE-30 (Qiagen). This allowed expression of the C-terminal portion of both kinesins fused to an N-terminal 6×His tag. Expression of recombinant proteins was induced in M15[Rep4] *Escherichia coli* (Qiagen) and proteins were subsequently isolated from cleared, sonicated bacterial lysates by nickel-affinity chromatography by standard methods. 2 mg of each recombinant was used to as immunogen in rabbits. 20 ml of each reactive antiserum were purified by binding to ∼5 mg of the respective recombinant protein coupled to CNBr-activated sepharose beads (Amersham), washed extensively with PBS and elution with first 0.2 M glycine, pH 3, then 0.2 M triethanolamine, pH 9. Affinity-purified polyclonal antibodies were dialysed against PBS and concentrated by ultrafiltration (Amicon Ultra 50k, Millipore). SDS-PAGE and immunoblotting were performed by standard methods, using polyclonal antibodies 1∶10 000 in 1% v/v skimmed milk in TBS-T (0.05% v/v Tween-20, 25 mM Tris-HCl, 150 mM NaCl, pH 7.5), followed by 1∶5 000 horseradish peroxidase-conjugated goat anti-rabbit immunoglobulins (Dako).

### Immunolocalisation and fluorescence *in situ* hybridisation

For analysis of localisation of GFP-tagged proteins or immunolocalisation in fixed whole cells, procyclic trypanosomes were harvested from mid-log phase cultures by centrifugation, washed once in PBS and allowed to adhere to ethanol-washed plain glass slides for 5 min in PBS (∼2×10^7^ cell ml^−1^). Cells were fixed for 5 min in 2% w/v formaldehyde in PBS, followed by permeabilisation in −20°C methanol for 15 min and rehydration in PBS. For the analysis of GFP fluorescence alone, samples were then mounted in a solution containing DAPI and a photostabilising agent (1% w/v 1,4-Diazabicyclo[2.2.2]octane, 90% glycerol, 50 mM sodium phosphate pH 8.0, 0.25 µg ml^−1^ 4′,6-diamidino-2-phenylindole). For immunolocalisation, samples were incubated for 1 h with either a 1∶10 dilution of KMX-1 hybridoma culture supernatant [67] or 1∶1000 dilution of affinity-purified anti-KIN13-1 or anti-KIN13-3 polyclonal antibodies in PBS. Slides were subsequently washed extensively, then incubated with 1∶200 TRITC-conjugated goat anti-mouse IgG (Stratech) or 1∶2 000 Alexa Fluor 594-conjugated goat anti-rabbit IgG (Invitrogen), respectively, and mounted as above.

For cytoskeleton preparations, cells settled as above were detergent extracted by the addition of 0.2% v/v Igepal CA-630 (Sigma-Aldrich) in PEME buffer (100 mM PIPES, pH 6.9, 2 mM EGTA, 1 mM MgSO_4_, 0.1 mM EDTA) for 2 min. Cytoskeletons were then fixed for 5 min in 2% w/v formaldehyde/PBS, followed by washing twice in PBS. Immunolocalisation was preformed as above.

For combined immunolocalisation and fluorescence *in situ* hybridisation, biotinylated 177-bp repeat was amplified by PCR using the primers GCG AAT TCT AAA TGG TTC TTA TAC GAA TG and TAC GAA GCT TAA CAC TAA AGA ACA GCG TTG in the presence of biotin-16-dUTP (Roche). Cells settled on glass slides as above were fixed for 10 min with 4% (w/v) formaldehyde, 5% (v/v) acetic acid in PBS and then permeabilized for 5 min in 0.1% v/v Igepal CA-630 in PBS. After washing, immunolocalisation was performed using the KMX-1 antibody as above, with the addition of 100 µg ml^−1^ DNase-free RNase A to the primary antibody (Sigma-Aldrich). Slides were then post-fixed in 3% formaldehyde in PBS for 10 min and washed in 0.25% glycine in PBS. Cells were incubated in hybridization buffer (50% v/v formamide, 5% w/v dextran sulphate, 25 mM sodium phosphate, 150 mM NaCl, 15 mM sodium citrate, pH 7.0) for 15 min at 40°C. Then, hybridization buffer containing 50 ng of probe DNA, 12.5 µg of herring sperm DNA (Roche) and 12.5 µg of yeast tRNA (Sigma-Aldrich) was added per sample. The slides were heated to 80°C for 5 min and then hybridised overnight at 37°C. The slides were washed twice for 10 min in 50% formamide, 2×SSC (300 mM NaCl, 30 mM sodium citrate, pH 7.0) at 37°C, for 10 min in 2×SSC at 55°C, and twice for 20 min in 0.2×SSC at 55°C, before transferring to PBS. Probe was detected using 2 µg ml^−1^ Alexa Fluor 488-conjugated streptavidin (Invitrogen).

For wide-field 3D deconvolution microscopy, cells were fixed for 10 min with 4% (w/v) formaldehyde, 5% (v/v) acetic acid in PBS and then permeabilized for 5 min in 0.1% Igepal CA-630 in PBS. After washing, immunolocalisation was preformed using the KMX-1 antibody and cells were mounted as above. Stacks of images encompassing 10–12 µm through z were captured on a Leica DM5500B microscope with motorised z-focus (100× objective, z-step of 194 nm), and deconvolved using the Iterative Deconvolve 3D plugin (Bob Dougherty, OptiNav Inc., Bellevue, WA) in the publicly-available ImageJ software (National Institute of Health USA; rsb.info.nih.gov). The deconvolution algorithm employed experimentally acquired point-spread functions, Wiener filter gamma = 0.001, low pass filter = 1 (x,y,z) and was run for a maximum of 128 iterations or until convergence of iterations to within 0.01% tolerance.

### Thin-section transmission election microscopy

Cells were fixed in solution with 2.5% w/v glutaraldehyde, post-fixed with 1% w/v osmium tetroxide, en bloc stained with 2% w/v aqueous uranyl acetate, dehydrated through acetone and embedded in epoxy resin. Ultrathin (∼75 nm thick) sections were stained with uranyl acetate and lead citrate and examined in a FEI Tecnai 12 electron microscope.

## Supporting Information

Figure S1 **Alignment of the motor domains from selected Kinesin-13 motors.** Sequences were aligned using MAFFT [59] (‘E-INS-I’ algorithm) followed by manual editing. The KVD and KEC motifs characteristic of Kinesin-13 sequences [25]; [26] are highlighted (red boxes). KIF18A and Klp67A (Kinesin-8) and KIFC1 and Ncd (Kinesin-14) are included for comparison. Prefixes: Arath: *Arabidopsis thaliana*; Chlre: *Chlamydomonas reinhardtii*; Drome: *Drosophila melanogaster*; Giala: *Giardia lamblia*; Homsa: *Homo sapiens*; Tetth: *Tetrahymena thermophila*; Trybr: *Trypanosoma brucei*.
(TIF)

Figure S2 **The specificity of affinity-purified rabbit polyclonal antibodies raised against TbKIN13-1 and TbKIN13-3.** Panels show detection of 10, 1 or 0.1 ng of the recombinant protein fragment used as immunogen (recomb.) or native protein in lysates of 5×10^6^ procyclic-form (PCF) or bloodstream-form (BSF) cells. 10 ng of unrelated recombinant is also show (first lane of each panel).
(TIF)

Figure S3 **Knockdown of** ***TbKIN13-1*** **results in persistent spindle-like structures in the cytoplasm of cells.** Panels show z-slices from 3D stacks of images captured by wide-field 3D deconvolution microscopy of fixed cells stained with the anti-β-tubulin monoclonal antibody KMX-1 [67] (red) and the DNA stain, DAPI (blue). Slices are spaced by 0.78 µm in z, and move from the slide surface towards coverslip, left to right. An average intensity projection of all slices (z-projection) and an overlay of the DAPI projection and phase contrast images (DAPI+phase) is also shown for each cell. Cells shown are from cultures at 48 h post-induction of RNAi against *TbKIN13-1*.
(TIF)

Figure S4 **Spindle microtubules in the nuclei of cells in following knockdown of** ***TbKIN13-1*** **.** Panels show thin-section transmission electron microscopy images demonstrating the presence of spindle microtubules in the nuclei of cells from cultures at 24 h post-induction of RNAi against *TbKIN13-1*. Nuclei frequently show parallel bundles of microtubules and distension of the nuclear envelope. The bottom-right image shows microtubules in transverse section (white box). The electron dense nucleoli (N) do not disassemble at mitosis in trypanosomes.
(TIF)

Table S1 **PCR primers used in the creation of endogenous-locus tagged GFP chimeras of** ***Trypanosoma brucei*** **Kinesin-13 proteins.** These oligonucleotides were used to create pEnG0-based tagging constructs as described in Materials and Methods.
(PDF)
