# Supplementary figures and images for: The Expanded Kinesin-13 Repertoire of Trypanosomes Contains Only One Mitotic Kinesin Indicating Multiple Extra-Nuclear Roles
Source: PLoS One. 2010 Nov 23;5(11):e15020. doi: 10.1371/journal.pone.0015020 (PMC2990766; doi:10.1371/journal.pone.0015020)

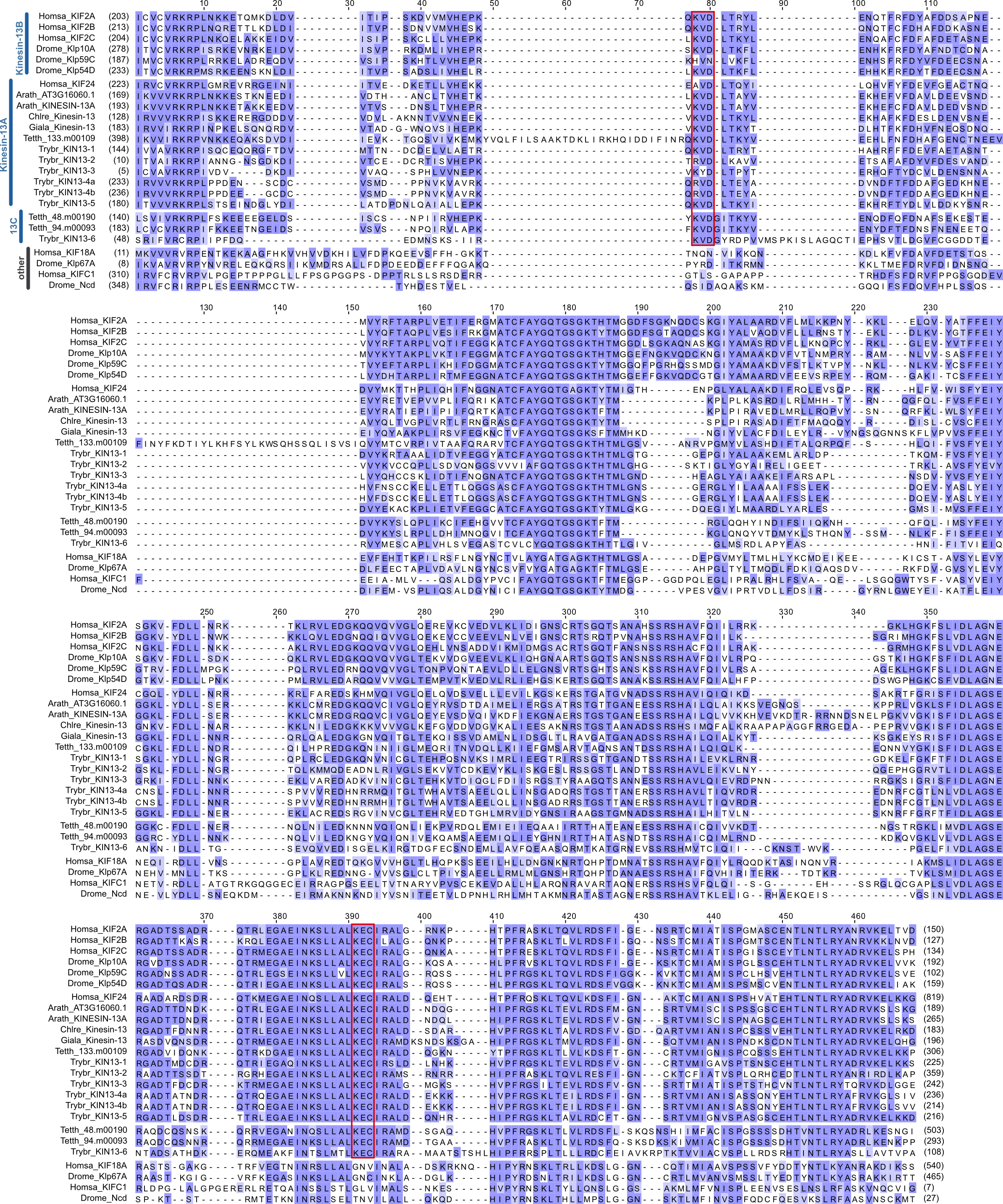

Supplement: Figure S1 — Alignment of the motor domains from selected Kinesin-13 motors. Sequences were aligned using MAFFT [59] (‘E-INS-I’ algorithm) followed by manual editing. The KVD and KEC motifs characteristic of Kinesin-13 sequences [25]; [26] are highlighted (red boxes). KIF18A and Klp67A (Kinesin-8) and KIFC1 and Ncd (Kinesin-14) are included for comparison. Prefixes: Arath: Arabidopsis thaliana; Chlre: Chlamydomonas reinhardtii; Drome: Drosophila melanogaster; Giala: Giardia lamblia; Homsa: Homo sapiens; Tetth: Tetrahymena thermophila; Trybr: Trypanosoma brucei. (TIF) [file pone.0015020.s001.tif]

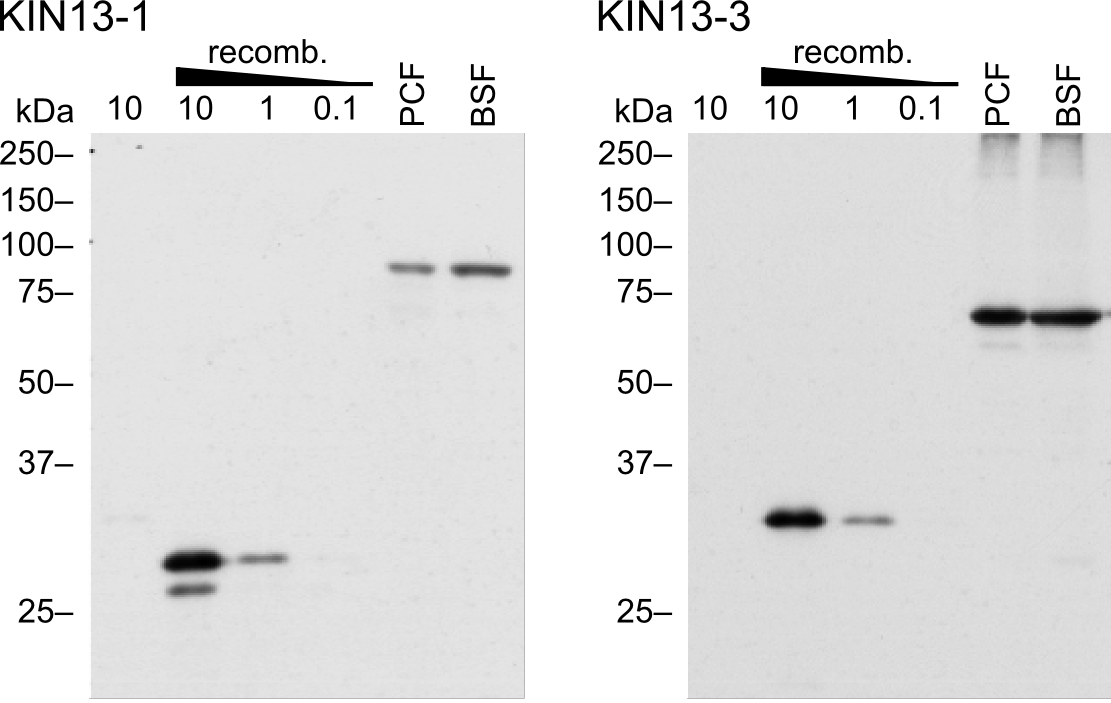

Supplement: Figure S2 — The specificity of affinity-purified rabbit polyclonal antibodies raised against TbKIN13-1 and TbKIN13-3. Panels show detection of 10, 1 or 0.1 ng of the recombinant protein fragment used as immunogen (recomb.) or native protein in lysates of 5×10^6^ procyclic-form (PCF) or bloodstream-form (BSF) cells. 10 ng of unrelated recombinant is also show (first lane of each panel). (TIF) [file pone.0015020.s002.tif]

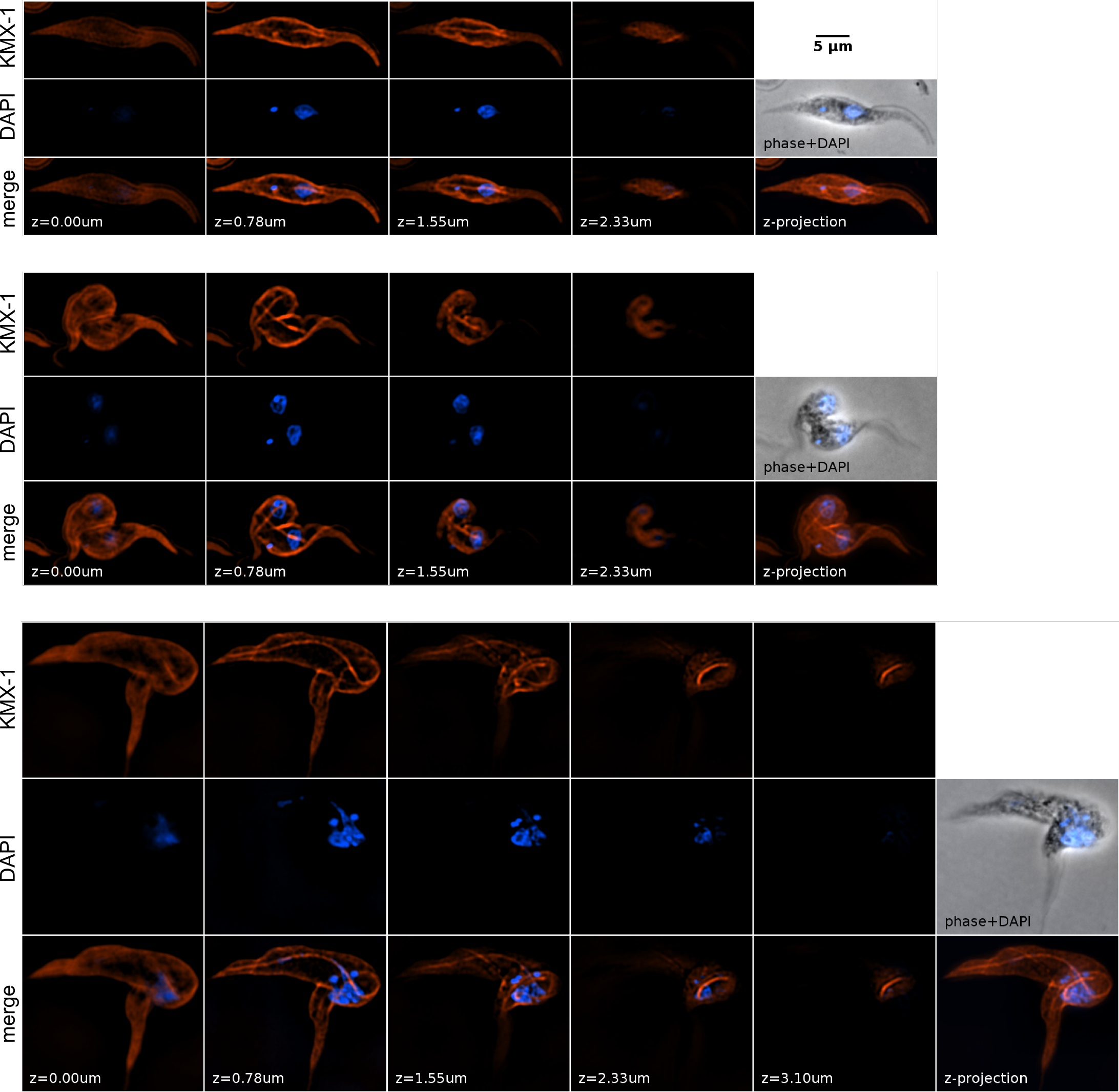

Supplement: Figure S3 — Knockdown of TbKIN13-1 results in persistent spindle-like structures in the cytoplasm of cells. Panels show z-slices from 3D stacks of images captured by wide-field 3D deconvolution microscopy of fixed cells stained with the anti-β-tubulin monoclonal antibody KMX-1 [67] (red) and the DNA stain, DAPI (blue). Slices are spaced by 0.78 µm in z, and move from the slide surface towards coverslip, left to right. An average intensity projection of all slices (z-projection) and an overlay of the DAPI projection and phase contrast images (DAPI+phase) is also shown for each cell. Cells shown are from cultures at 48 h post-induction of RNAi against TbKIN13-1. (TIF) [file pone.0015020.s003.tif]

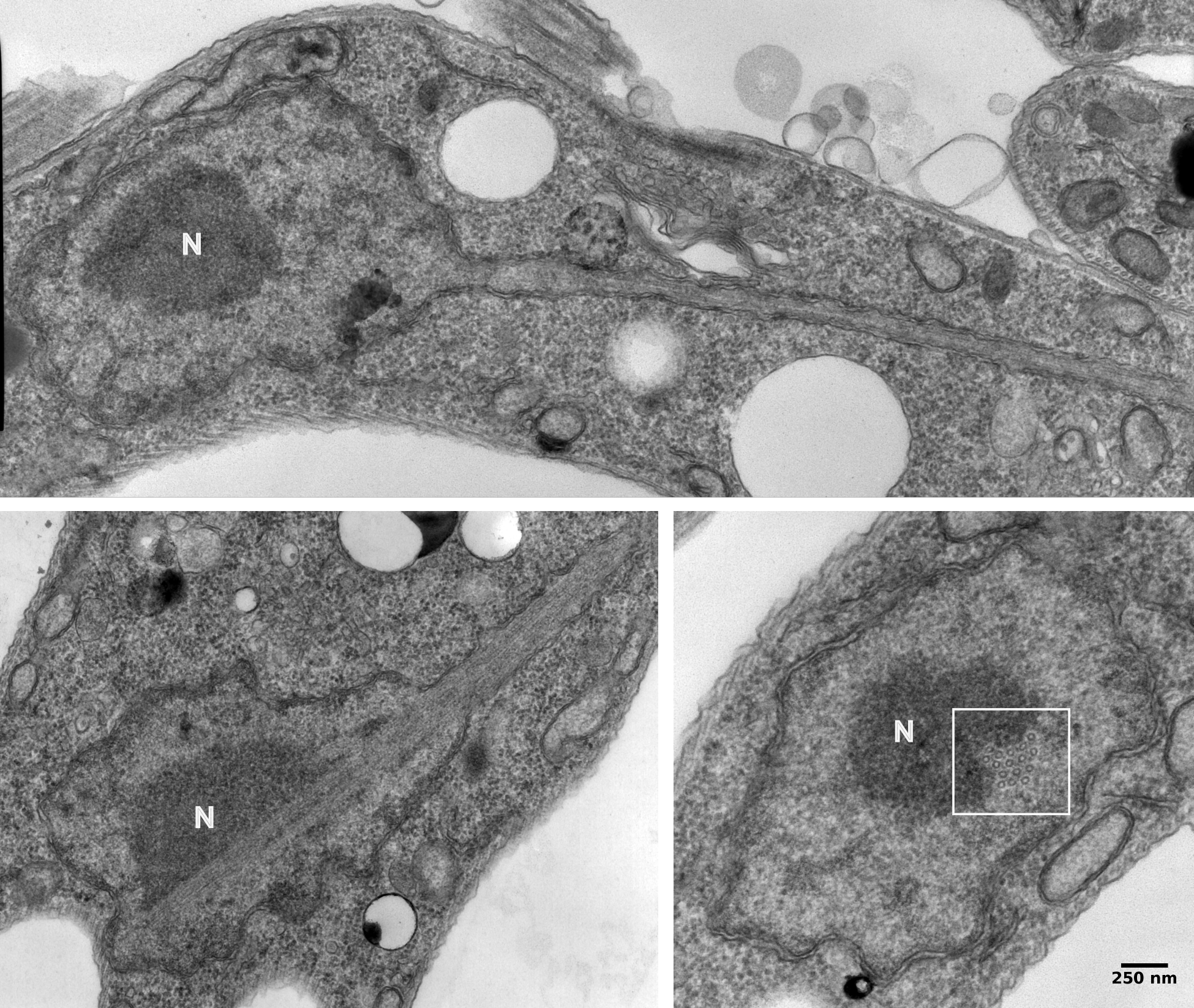

Supplement: Figure S4 — Spindle microtubules in the nuclei of cells in following knockdown of TbKIN13-1 . Panels show thin-section transmission electron microscopy images demonstrating the presence of spindle microtubules in the nuclei of cells from cultures at 24 h post-induction of RNAi against TbKIN13-1. Nuclei frequently show parallel bundles of microtubules and distension of the nuclear envelope. The bottom-right image shows microtubules in transverse section (white box). The electron dense nucleoli (N) do not disassemble at mitosis in trypanosomes. (TIF) [file pone.0015020.s004.tif]
