# Supplementary material for: The Expanded Kinesin-13 Repertoire of Trypanosomes Contains Only One Mitotic Kinesin Indicating Multiple Extra-Nuclear Roles
Source: PLoS One. 2010 Nov 23;5(11):e15020. doi: 10.1371/journal.pone.0015020 (PMC2990766; doi:10.1371/journal.pone.0015020)
Supplement: Table S1 — PCR primers used in the creation of endogenous-locus tagged GFP chimeras of Trypanosoma brucei Kinesin-13 proteins. These oligonucleotides were used to create pEnG0-based tagging constructs as described in Materials and Methods. (PDF) [file pone.0015020.s005.pdf]

Supplemental Table S1. PCR primers used in the creation of endogenous-locus tagged GFP chimeras of *Trypanosoma brucei* Kinesin-13 proteins.

Protein	GeneID	3'UTR targeting sequence		CDS targeting sequence		3' intergenic region	
		forward	reverse	forward	reverse	forward	reverse
KIN13-1	Tb09.160.2260	catctaga<br>tgggggagggaagaaa	tagcggccgc<br>ggcaggaagtgatgtacaaa	tagcggccgc<br>ggcactgataaaggaggag	caggatcc<br>aatcccgttttgctcga	caggatcc<br>tgggggagggaagaaa	cagagctc<br>ttgccgatgataccacca
KIN13-2	Tb11.02.2260	catctaga<br>acctgcaaattaaatcacatgc	tagcggccgc<br>aagcgttaccctacgc	tagcggccgc<br>ttggaggtgaaggttgtg	caggatcc<br>cacgctttcaagttcatgaag	caggatcc<br>acctgcaaattaaatcacatgc	cagagctc<br>atttccctccaagacacg
KIN13-3	Tb11.02.2970	catctaga<br>cgagctttcttcaatttcaatgc	tagcggccgc<br>agtcagaactccagaatatgtg	tagcggccgc<br>taaggatgagatacgtgcc	caggatcc<br>attgcgtctcttcatggaag	caggatcc<br>cgagctttcttcaatttcaatgc	cagagctc<br>attatctctccctcaaacgc
KIN13-4a	Tb927.4.3910	catctaga<br>tacgagaaagccgacgta	tagcggccgc<br>aggatggggaaccacaa	tagcggccgc<br>atgaaagaactagaggaccg	caggatcc<br>caccggtcgaattgctt	(as targeting seq)	cactcgag<br>aagttgttgaagagtagaggc
KIN13-4b	Tb927.8.8350	catctaga<br>acacaatgcaacatcaacc	tagcggccgc<br>ggtgggaagatgttgga	tagcggccgc<br>ggcagttagaagatcgtgt	caggatcc<br>gttttgttgctgttgtatttgacg	(as targeting seq)	cactcgag<br>gtcgaggaaatatattgtcttgg<br>a
KIN13-5	Tb11.02.0790	catctaga<br>atgagcttccctaccg	tagcggccgc<br>ccttctcaacttctatacagcc	tagcggccgc<br>aggataataccattctgggc	caggatcc<br>agactccaggcggttta	(as targeting seq)	cactcgag<br>gtttcgttttcgatgttcgg
KIN13-6	Tb09.211.1400	catctaga<br>tttttatcggttcgttctccg	tagcggccgc<br>aaaaagagcacattgacgc	tagcggccgc<br>ctgaaggagatggtcgt	caggatcc<br>cctctgcgtagcacac	(as targeting seq)	cactcgag<br>agccacacttggacctta
PFR1	Tb927.3.4300	catctaga<br>tgtggccgcaattattatgta	tagcggccgc<br>tgatgaagaaaagtgtgagca	tagcggccgc<br>ttcaaccaccctgtgga	caggatcc<br>cggtgcagtgttgtttc	caggatcc<br>tgtggccgcaattattatgta	cagagctc<br>aggataatcgatttcttgtgttga
